# Oxidized LDL-induced JAB1 influences NF-κB independent inflammatory signaling in human macrophages during foam cell formation

**DOI:** 10.1186/s12929-017-0320-5

**Published:** 2017-02-07

**Authors:** Anja Schwarz, Gabriel A. Bonaterra, Hans Schwarzbach, Ralf Kinscherf

**Affiliations:** Anatomy and Cell Biology, Department of Medical Cell Biology, University of Marburg, Robert-Koch-Straße 8, 35032 Marburg, Germany

**Keywords:** Atherosclerosis, JAB1, Macrophages, oxLDL, Foam cells

## Abstract

**Background:**

Oxidized low-density lipoprotein (oxLDL) mediates the transformation of macrophages (MΦ) to cholesterol-rich foam cells and the release of pro-inflammatory cytokines during atherogenesis. JAB1 (Jun activation domain binding protein-1) is present in all stages of human plaques, involved in the Toll-like receptor-mediated activation of p38 mitogen-activated protein kinase (MAPK) and controls nuclear factor-kappa B (NF-κB) activation. Thus, we were interested in the role of JAB1 during foam cell formation of MΦ after oxLDL exposition.

**Methods and results:**

We found that JAB1 was present in CD68-immunoreactive (−ir) MΦ in atherosclerotic plaques of apolipoprotein E knockout (ApoE^−/−^) mice after a high cholesterol/fat diet. Furthermore, differentiated human U937 MΦ - incubated with oxLDL (4 h) to induce foam cell formation – showed a significant increase of JAB1 (50 μg/ml: 1.39 + 0.15-fold; 100 μg/ml: 1.80 + 0.26-fold; 200 μg/ml: 2.05 + 0.30-fold; *p* < 0.05) on the protein level compared to the control. Independent from JAB1 silencing, we found an increase of total cholesterol (TC), free cholesterol (FC) and cholesteryl ester (CE) after oxLDL exposition. However, siJAB1-MФ showed a reduction of tumor necrosis factor-alpha (TNF-α) (36%; *p* < 0.05 vs. non-transfected MФ) and interleukin (IL)-6 (30%; *p* < 0.05 vs. non-transfected MФ) mRNA expression, as well as TNF-α (46%; *p* < 0.05 vs. non-transfected MФ) and IL-6 (32%; *p* < 0.05 vs. non-transfected MФ) protein secretion after oxLDL exposition. In parallel with an upregulation of inflammatory cytokines (TNF-α, IL-6) after oxLDL exposition, we found a significant (*p* < 0.05) increase of 37% in p38 MAPK activation after 4 h oxLDL-treatment, independent from NF-kB signaling. In this context, we showed regional co-localization of JAB1 with p38 MAPK in atherosclerotic plaques of ApoE^−/−^ mice. Moreover, we detected interaction of JAB1 with p38 MAPK in U937 cells.

**Conclusion:**

We demonstrate that oxLDL induces JAB1 expression and influences its cellular localization, whereby the p38 MAPK signaling pathway is modified with consequences for inflammation of human MΦ in foam cells and atherosclerotic lesions.

**Electronic supplementary material:**

The online version of this article (doi:10.1186/s12929-017-0320-5) contains supplementary material, which is available to authorized users.

## Background

Atherosclerosis is a chronic and inflammatory disease, and it is well established that atherosclerotic lesions arise from an enhancement of LDL uptake by monocytes and macrophages (MΦ) [1]. The interaction of oxLDL and MΦ is a hallmark in the development and progression of atherosclerosis [2]. MΦ which internalized oxLDL via scavenger receptors subsequently transform into lipid-laden foam cells [3] and release various pro-inflammatory cytokines such as tumor necrosis factor-alpha (TNF-α) - under the transcription control of NF-κB - upon exposure to oxLDL [4].

Thus, NF-κB is a key regulator of inflammation during the atherosclerotic process. Under normal conditions, Ik-Bα (nuclear factor of kappa light polypeptide gene enhancer in B-cells inhibitor, alpha) binds to NF-κB, a complex which remains in the cytosol when inactive. Various exogenous inflammatory signals lead to phosphorylation of Ik-Bα on the Ser32/Ser36 residues by IkBkinaseß (IKKß). The phosphorylated Ik-Bα degrades rapidly via the ubiquitin proteasome system (UPS) [5]. Thereby, NF-κB molecules can translocate into the nucleus to allow gene transcription. NF-κB activation controls the Ik-Bα expression with an auto-regulatory negative feedback loop [6].

Equally, oxLDL activates the mitogen-activated protein kinase (MAPK) signaling transduction pathway. Most recent studies have shown that an inhibition of p38 MAPK restrains the oxLDL-induced foam cell formation in murine RAW264.7 cells [7]. The direct substrate of p38 MAPK is the MAPK-activated protein kinase-2 (MK2). Deletion of the MK2 gene in mice resulted in an impaired inflammatory response with reduced production of pro-inflammatory mediators (TNF-α, IL-6) in response to a challenge with lipopolysaccharide (LPS) [8, 9].

Furthermore, it was supposed that an increased expression of JAB1 correlates with athero-progression [10], because Constitutive photomorphogenesis 9 (COP9) signalosome complex unit 5 (CSN5) was found in endothelial cells and MΦ of human atherosclerotic lesions [10–12] and is involved in the regulation of protein stability and protein degradation [13–15]. Especially, JAB1 was implicated in the ubiquitin-proteasome degradation machinery and the regulation of signal transduction pathways [16]. In this context, the deneddylation activity was pinpointed to the JAB1/MPN (Mpr/Pad1/N-terminal) domain metalloenzyme (JAMM) submotif located in the MPN domain of JAB1 [17]. Also, it was shown that JAB1 controls the Ik-Bα stability which regulates the activity of SCF (Skp1, Cullins, F-box proteins)-type CRL (Cullin-RING ubiquitin Ligase) by deneddylation [18, 19]. JAB1 silence induced Ik-Bα degradation, enhanced NF-κB activity, increased the TNF-a-induced expression of adhesion molecules (CCL2, ICAM-1, VCAM-1) and induced monocytes arrest rate on inflamed endothelium *in vitro* [10]. Furthermore, JAB1 is a critical factor to trigger an appropriate innate immune response and is required for activation of p38 MAPK [20]. Deng et al. showed that JAB1 deletion in bone marrow-derived MΦ from CSN5-deficient mice attenuated significantly the TNF-α-mediated induction of p38 MAPK phosphorylation and increased the expression of anti-inflammatory genes [20].

The purpose of our investigation was to find out if Jab1 expression is regulated by oxLDL as well as its relation to the NF-kB transcription factor and the activation of the p38 MAPK signaling pathway during foam cell formation.

## Methods

### Animals

Male B6.apolipoprotein E knockout (ApoE^−/−^) B6.129P2-Apo^tm1Unc^/J mice (Charles River, Sulzfeld, Germany) were used for experiments (*n* = 4). At an age of 10 weeks, the offspring was fed with an adjusted-calories cholesterol-enriched diet (CED, TD.88137; Harlan Teklad, Madison, WI) for a period of 20 weeks. The brachiocephalic trunks were removed, shock-frozen in liquid nitrogen–cooled isopentane and kept at −70 °C in a freezer until used for immunohistochemical investigations [21]. All animal experiments were approved by the Regierungspräsidium Karlsruhe and the local authorities at the Universitiy of Heidelberg (Az 35–9185.81/g-99/06).

### Cell culture, transfection and gene silencing

The human leukemic monocyte lymphoma cell line U937 (Cell line service, Eppelheim, Germany) was used. Originally, the culture was derived from a histiocytic lymphoma and is frequently used as a model of monocyte/MΦ cell lineage [22, 23]. U937 cells were differentiated into MΦ using 20nM Phorbol 12-mystriate 13-acetate [PMA, (Sigma-Aldrich Chemie GmbH Munich, Germany)] in RPMI 1640 medium for 24 h. Afterwards, MΦ were incubated with human oxLDL (50–200 μg/ml) or LPS (0.1 μg/ml) for 4 h. Transfection of U937 MФ with 50nM pre-designed JAB1 siRNA (siJAB1-MФ) (FlexiTube GeneSolutionGS10987, QIAGEN GmbH, Hilden, Germany) and negative siRNA (nsiJAB1-MФ) (AllStars Negative Control, QIAGEN GmbH, Hilden, Germany) was performed using HiPerfect Transfection Reagent (QIAGEN GmbH, Hilden, Germany) following the manufacturer’s instruction. Therefore, cells incubated with “medium” containing HiPerfect Transfection Reagent was used as additional control. After transfection, cells were allowed to recuperate for 48 h before being treated with 50 μg/ml oxLDL (4 h). As positive control, we used the AllStars Hs Cell Death Control siRNA (QIAGEN GmbH, Hilden, Germany), a compound of highly potent siRNAs targeting ubiquitously expressed human genes that are essential for cell survival. Transfection efficiency was estimated by observing cells by light microscopy 48 h after transfection with the AllStars Hs Cell Death Control siRNA.

### SDS-PAGE and western blot

PMA-differentiated U937 MΦ were washed in ice-cold phosphate buffered saline (PBS) and lysed using RIPA (radioimmuniprecipitation assay) buffer pH 7.5 (Cell Signaling Technology Europa, Leiden, The Netherlands), containing protease/phosphatase inhibitor cocktail (Cell Signaling Technology, Boston, USA). The protein concentrations were determined spectrophotometrically using the Pierce BCA (bicinchoninic acid) Protein Assay (Thermo Scientific, Rockford, USA). Proteins were loaded on NuPAGE® Novex® 4–12% Bis-Tris Gels, pre-cast polyacrylamide gels (Life Technologies GmbH, Darmstadt Germany). Proteins were transferred onto 0.45 μm nitrocellulose membranes [Millipore (Billerica, MA, USA)]. Primary Antibodies (see Additional file 1) were added and incubated overnight at 4 °C in blocking buffer (5% milk). Membranes were incubated with enhanced ECL-anti-goat IgG-POD antibody, ECL-anti-mouse IgG-POD antibody or ECL-anti-rabbit IgG-POD antibody. The peroxidase reaction was visualized by AceGlow chemiluminescence substrate (Peqlab, Erlangen, Germany) and documented by the Fusion-SL Advance™ imaging system (Peqlab) according to the instruction manual. The intensity of the specific western blot bands was quantified using the software ImageJ from the National Institutes of Health (Bethesda, USA).

### LDL oxidation

LDL (Cell sciences, Canton, USA) oxidation was performed as previously described by Galle and Wanner [24] and Steinbrecher [25]. The LDL was resuspended in endotoxin-free PBS (LONZA, Ratingen, Germany) to a final concentration of 1 mg protein/ml and dialyzed using Slide-A-Lyzer Dialysis Cassettes 7 K MWCO (Thermo Fisher Scientific Inc., Rockford, USA). The grade of oxidation was verified by spectrophotometric analysis (absorbance spectrum between 400 and 600 nm). After oxidative modification, the absorption peaks at at 460 and 485 nm, which are characteristic for native LDL, disappear [24].

### Viability assay

For analysis of cell survival, U937 (6.25*10^5^ cells/ml) cells were cultured in 96 well plates, differentiated into MΦ as described above and treated with or without oxLDL or LPS. Cell viability was assessed using PrestoBlue™ [26], according to the manufacturer’s protocol. 1 h after the addition of PrestoBlue™, the optical density (OD) was measured at 570 nm and 600 nm (as reference) by a SUNRISE ELISA-reader (Tecan, Salzburg, Austria). Results are presented in % survival = [Sample OD_(570nm - 600nm reference)_*100)/Control OD_(570nm - 600nm reference)_]. As control (=100% were cultured with medium alone (i.e. without addition of test substances) [26].

### OilRed O staining

The Oil Red O [ORO, (Sigma-Aldrich)] working solution was prepared by diluting the stock solution (3 mg/ml ORO dissolved in 2-propanol) with distilled water (3:2). For staining, PMA differentiated U937 MΦ were fixed in 10% PFA, and the ORO working solution was added to the culture dishes afterwards. The nuclei were stained using Mayer’s *hemalum* solution (Carl Roth, Karlsruhe, Germany).

### Cholesterol/cholesteryl ester quantitation

For analyses of the TC and FC, U937 cells (10^6^ cells/well) were cultured in 6 well plates, differentiated into MΦ as described above and incubated with or without 50 μg/ml oxLDL for 4 h. TC and FC were determined using the Cholesterol/Cholesteryl Ester Quantitation Assay (Abcam plc., Cambridge, UK), according to the manufacturer’s protocol. The OD was measured at 570 nm by a SUNRISE ELISA-reader (Tecan, Salzburg, Austria). TC and FC results are presented in μg/mg protein (determined as described above). CE was determined by subtracting the value of FC from the TC.

### Extraction of cytoplasmic and nuclear proteins

After treatment, PMA-differentiated U937 MΦ were washed in ice-cold PBS and lysed by cytoplasmic extract buffer (10 mol/ml HEPES (pH 7.9), 10 mol/ml KCl, 0.1 mol/ml EDTA, 0.3% NP-40) (Roth) in the presence of protease/phosphatase inhibitor cocktail (Sigma Aldrich) and centrifuged (1,500 x g; 5 min; 4 °C). The supernatant containing the cytoplasmic fraction was separated. The pellet was suspended in RIPA buffer and centrifuged to obtain the supernatant containing the nuclear fraction.

### Co-immunoprecipitation assay

Protein interactions were verified for U937 MΦ by using the Thermo Scientific™ Pierce™ Co-immunoprecipitation (Co-IP) Kit (Thermo Fisher Scientific Inc., Rockford, USA). The immunoprecipitation of the endogenous protein occurred with JAB1 antibody-coupled resin. For that, the cells were lysed with Lysis/Wash buffer supplemented with a protease/phosphatase inhibitor cocktail (Sigma Aldrich) and centrifuged to pellet the cell debris. 1 mg of total protein from the supernatant was clarified using the Control Agarose Resin Column (crosslinked 4% beaded agarose) to reduce nonspecific protein binding. The eluate of the Control Agarose Resin served as negative control for unspecific protein binding to resin, because the supplied Pierce Control Agarose Resin is composed of the same support material as the AminoLink Plus™Coupling Resin, but was not amine-reactive. The co-immunoprecipitation was performed according to the manufacturer’s instructions followed by a western blot using corresponding antibodies of interest.

### Immunocyto−/−histofluorescence confocal laser scanning microscopy

Tissue sections or cells were fixed with ice-cold methanol and permeabilized with 1% Triton-X 100 in PBS. Thereafter, the detergent was removed by repeated washing in PBS. Primary antibodies (see Additional file 1) were applied in PBS overnight (4 °C). After the incubation with secondary antibodies (see Additional file 1) and subsequent staining with DAPI, cells were covered with IMMU-MOUNT (Thermo Electron Corporation; Pittsburgh; USA) and a glass coverslip. Images were taken by confocal laser scanning microscopy (Nikon Eclipse Ti-E, Düsseldorf, Germany).

### Reverse transcription and quantitative polymerase chain reaction

Total RNA was extracted from human U937 MФ using PeqGold TRIFast™ from Peqlab (Erlangen, Germany). DNase I (RNase-free; Thermo Scientific) was used according to the manufacturer’s instructions. The AffinityScript Multiple Temperature Reverse Transcriptase and the Brilliant III Ultra-Fast SYBR® Green Master Mix were obtained from Agilent Technologies (Waldbronn, Germany). The QuantiTect Primer Assays were purchased from QIAGEN GmbH (Hilden, Germany) (see Additional file 2). RNA concentration and purity were determined by absorbance measurements at 260 nm and 280 nm (A260/A280 = 1.7–2.0) using a NanoDrop 8000 Spectrophotometer (Thermo Scientific, Schwerte, Germany). Total RNA integrity was confirmed by lab-on-a-chip technology, using an RNA 6000 NanoChip kit on an Agilent 2100 Bioanalyzer (Agilent Technologies, Waldbronn, Germany). On average, we obtained a RNA Integrity Number (RIN) of 9.71 ± 0.03 (SEM). 1 μg of template RNA was used for cDNA synthesis; RNA was reverse transcribed using Oligo(dT)_12–18_ primer and the AffinityScript™ Multiple Temperature Reverse Transcriptase, according to the manufacturer’s instructions. The cDNA (diluted 1:20) was amplified using the Brilliant III Ultra-Fast SYBR® Green QRT-PCR Master Mix (Stratagene-Agilent Technologies, Waldbronn, Germany). Amplification and data analyses were performed using the Mx3005P™ QPCR System (Stratagene). The data were analyzed using the relative standard curve method. The NormFinder software program was used to ascertain the most suitable reference gene to normalize the RNA input as described earlier [27].

### Statistical analyses

Statistical analyses were performed using SigmaPlot 12 (Systat Software Inc., USA). After testing for normality (by Shapiro-Wilk), the unpaired Student’s *t*-test or one-way analysis of variance (ANOVA) was used. Data are reported as mean ± standard error of the mean (SEM). *P* < 0.05 was considered as statistically significant.

## Results

### Localization of JAB1 in CD68^+^ MΦ in atherosclerotic plaques of hypercholesterolemic ApoE^−/−^ mice

**Fig. 1:**
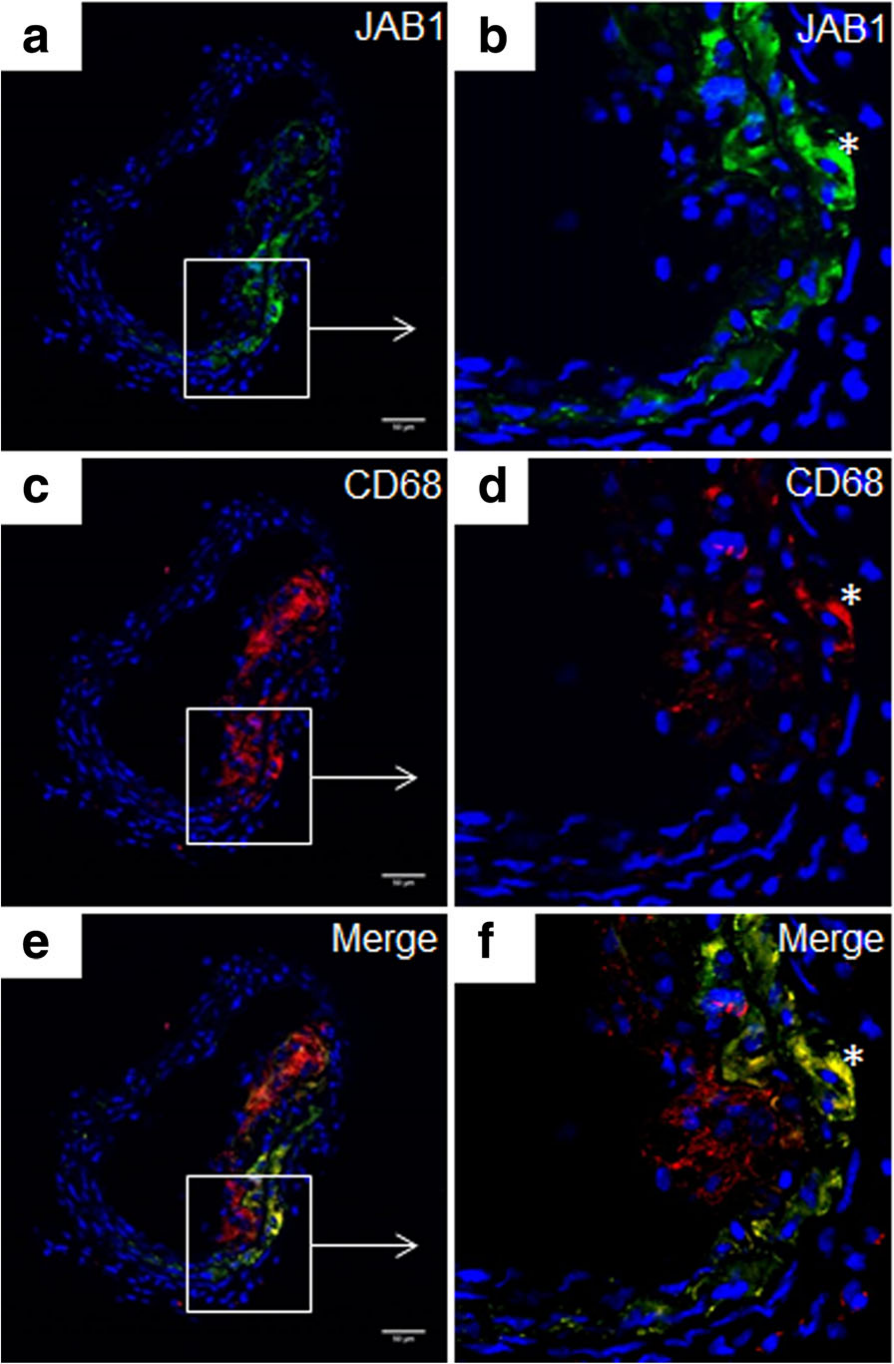
Localization of JAB1 in CD68^+^ MΦ in atherosclerotic plaques of hypercholesterolemic ApoE^−/−^ mice. Representative cross-sections of the brachiocephalic trunk of ApoE^−/−^ mice at 30 weeks of age are shown. In an atherosclerotic plaque of ApoE^−/−^ mice JAB1-immunoreactivity (**a**, **b**) was localized in CD68^+^ MΦ (**c**, **d**), as seen in merge (**e**, **f**) labelled by *. JAB1- and CD68-immunoreactivity was detected by confocal laser scanning microscopy (Nikon Eclipse) and analyzed with Fiji ImageJ software. Nuclei were counterstained with DAPI (blue). Representative photos from 4 independent experiments are shown. Bars: 50 μm

Our immunohistochemical investigations showed that JAB1-ir cells localize in atherosclerotic lesions of ApoE^−/−^ mice after 20 weeks of CED (Fig. 1). JAB1-ir cells (Fig. 1a, b) and CD68-ir MФ (Fig. 1c, d) were found predominantly in shoulder and deeper regions of the plaque, but not in non-atherosclerotic vessel wall segments e.g. of the brachiocephalic trunk. In detail, double immunostaining procedures revealed a co-localization of JAB1- and CD68-immunoreactivity in MФ in deeper regions of the plaque (Fig. 1e, f). However, not all CD68-ir MФ were positive for JAB1-ir (Fig. 1e, f).

### OxLDL influenced JAB1 protein expression and foam cell formation in differentiated human MΦ

**Fig. 2:**
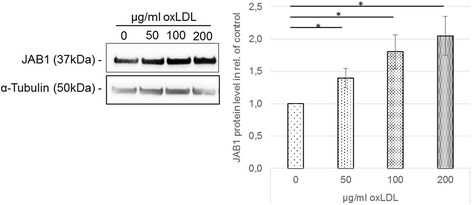
OxLDL influence JAB1 protein expression and foam cell formation in differentiated human MΦ. PMA-differentiated human U937 MΦ were incubated (4 h) with medium alone (= control) or oxLDL (50, 100, 200 μg/ml). The JAB1 and α-tubulin protein levels were determined using western blot analysis and quantified by ImageJ. Expression was normalized against α-tubulin. Bars represent mean ± SEM of 4 independent experiments. *p < 0.05 significance vs. control

MФ-derived foam cell formation is a hallmark of atherosclerosis. Since our immunohistochemical stainings of the brachiocephalic trunk of ApoE^−/−^ mice revealed a co-localization of JAB1-ir and CD68-ir MФ (Fig. 1e, f), we investigated the effect of oxLDL in PMA-differentiated human U937 MΦ on JAB1 protein expression and foam cell formation *in vitro*. We showed that oxLDL (50 μg/ml, 100 μg/ml, 200 μg/ml) exposition (4 h) induced foam cell formation as indicated by an increased inclusion of lipid droplets in the cytosol and the perinuclear area (see Additional file 3). LPS treatment that was performed as positive control for inflammation did not result in foam cell formation (see Additional file 3). However, the viability of human U937 MФ was unchanged after LPS or oxLDL exposition in comparison to the control (see Additional file 3). We next investigated, whether oxLDL influences the expression of JAB1 in differentiated human U937 MФ. We found that the protein level of JAB1 significantly and concentration-dependently increased after 4 h oxLDL exposition (50 μg/ml: 1.39 + 0.15-fold; 100 μg/ml: 1.80 + 0.26-fold; 200 μg/ml: 2.05 + 0.30-fold; *p* < 0.05) in comparison with the control (Fig. 2).

### JAB1 silencing attenuated the oxLDL-induced expression and secretion of TNF-α and IL-6 without influencing the cellular cholesterol

**Fig. 3:**
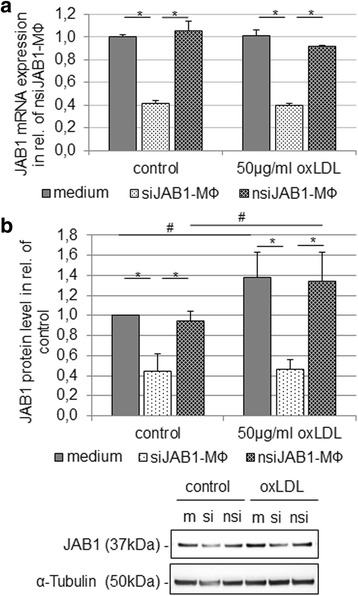
Knock-down of JAB1 mRNA and protein level in human MФ. PMA-differentiated human U937 MΦ were transfected with siRNA JAB1 (siJAB1-MФ) or negative siRNA (nsiJAB1-MФ) for 48 h. Non-transfected MФ, nsiJAB1-MФ and siJAB1- MФ were treated with or without (control) oxLDL (50 μg/ml) for 4 h. **a** The JAB1 mRNA expression levels were determined using RT-qPCR. Bars represent mean ± SEM of 3 independent experiments. *p < 0.05 significance vs. non-transfected MФ or nsiJAB1-MФ. **b** The JAB1 and α-tubulin protein levels were determined using western blot analysis and quantified by ImageJ. Expression was normalized against α-tubulin. Bars represent mean ± SEM of 5 independent experiments. [m] – non-transfected MФ, [si] - siJAB1-MФ, [nsi] – nsiJAB1-MФ; *p < 0.05 significance vs. non-transfected MФ or nsiJAB1-MФ, ^#^ *p* < 0.05 significance vs. control

Our finding that oxLDL induces foam cell formation with increased JAB1 protein expression suggests a functional effect of JAB1 in MФ after oxLDL exposition. Next, we transiently silenced *JAB1* expression in U937 MФ (siJAB1-MФ). Microscopic investigations revealed no morphological differences among non-transfected MФ (medium), nsiJAB1-MФ or siJAB1-MФ (see Additional file 4a-c). As negative control, we used a non-silencing siRNA (nsiJAB1-MФ) without homology to any known mammalian gene. The cell death control siRNA showed the efficient transfection of siRNA by cell death (see Additional file 4d). The transient transfection achieved a significant (*p* < 0.005) knock-down of JAB1 mRNA 61% or protein 60% compared to non-transfected MФ (Fig. 3a, b). After oxLDL exposition (4 h) the JAB1 expression showed no significant difference on the mRNA as well on the protein level in comparison to control in siJAB1-MФ (Fig. 3).

**Fig. 4:**
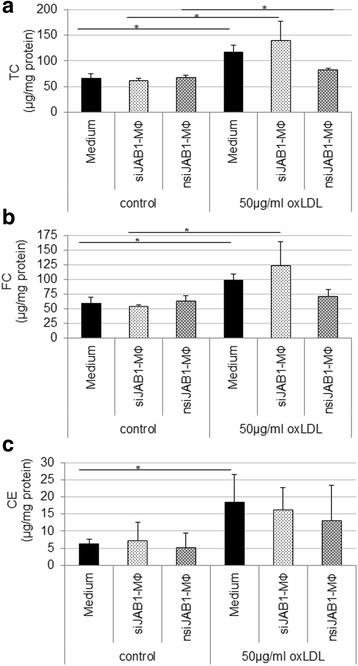
TC, FC and CE contents in non-transfected, siJAB1- or nsiJAB1- U937 MФ. Cells (1*10^6^ cells/well) were cultured in the presence of PMA (20nM) for 24 h and transfected with siRNA JAB1 (siJAB1-MФ) or negative siRNA (nsiJAB1-MФ) for 48 h. Non-transfected MФ, nsiJAB1-MФ or siJAB1- MФ were incubated (4 h) with oxLDL (50 μg/ml) or without (control). **a** TC, total cholesterol, (**b**) FC, free cholesterol and (**c**) EC, esterified cholesterol, were normalized to total protein. Data are expressed as mean + SEM of 4 independent experiments. *p < 0.05 significance vs. corresponding control

In order to test whether JAB1 plays a role during oxLDL uptake and intracellular cholesterol concentration, we measured the cholesterol content of siJAB1-MФ versus non-transfected MФ and nsiJAB1-MФ (Fig. 4). We examined the effect of *JAB1 silencing* on TC, FC and CE accumulation. At first, we show a significant (*p* < 0.05) increase of TC in non-transfected MФ (1.8-fold), nsiJAB1-MФ (1.2-fold) and siJAB1-MФ (2.3-fold) after oxLDL exposition in comparison with corresponding control MФ (Fig. 4a). Furthermore, FC and CE were increased in non-transfected MФ (FC: 1.7-fold *p* < 0.05; CE: 2.9-fold *p* < 0.05), nsiJAB1-MФ (FC: 1.1-fold; CE: 2.6-fold) and siJAB1-MФ (FC: 2.3-fold; *p* = 0.05; CE: 2.3-fold) after 4 h oxLDL exposition in comparison with corresponding control MФ (Fig. 4b, c). In summary, JAB1 silencing had no significant effects on TC, FC and CE accumulation after oxLDL exposition in comparison with non-transfected MΦ or nsiJAB1-MΦ.

**Fig. 5:**
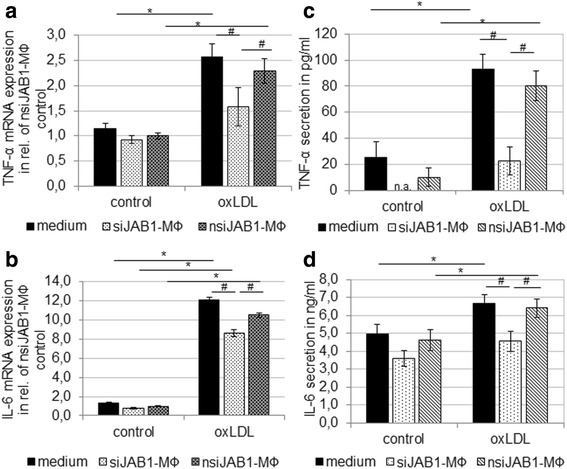
JAB1 silencing attenuates the oxLDL-induced expression and secretion of TNF-α and IL-6. PMA-differentiated human U937 MΦ were treated (4 h) with 50 μg/ml oxLDL and compared to the control (medium). TNF-α (**a**) and IL-6 (**c**) mRNA expression levels were determined using RT-qPCR. TNF-α (**b**) and IL-6 (**d**) protein secretion was determined using ELISA. Bars represent mean ± SEM of 3 independent experiments. *p < 0.05 significance vs. control, ^#^ *p* < 0.05 significance vs. non-transfected MФ or nsiJAB1-MФ

Since oxLDL induces production and release of pro-inflammatory cytokines, we examined the effect of JAB1 silencing on expression and secretion of TNF-α and IL-6. Here we observed a significant increase of mRNA expression (2.58-fold TNF-α, 12.15-fold IL-6) and protein secretion (67.74 pg/ml TNF-α, 1.7 ng/ml IL-6) after oxLDL exposition (4 h) in non-transfected MΦ (Fig. 5). In addition, after oxLDL exposition (4 h), nsiJAB1-MΦ showed a significant increase of mRNA expression (2.29-fold TNF-α, 10.53-fold IL-6) and protein secretion (80.35 pg/ml TNF-α, 6.39 ng/ml IL-6) in comparison with nsiJAB1-MΦ without oxLDL exposition (Fig. 5). Furthermore, JAB1 silencing resulted in a significant (*p* < 0.005) decrease of TNF-α and IL-6 mRNA expression (31% TNF-α; 18% IL-6) in comparison to nsiJAB1-MФ (as well as non-transfected MΦ) after oxLDL treatment (4 h) (Fig. 5a, b). Moreover, JAB1 silencing also resulted in a significant (*p* < 0.05) inhibition of TNF-α and IL-6 secretion (72% TNF-α; 29% IL-6) in comparison with nsiJAB1-MФ after oxLDL treatment (4 h) (Fig. 5c, d).

### OxLDL induced JAB1 expression by NF-κB-independent signaling in human MФ

After our demonstration that oxLDL leads to the expression of inflammatory molecules in dependence of JAB1 expression, we examined whether the oxLDL-induced expression of JAB1 influences pivotal proteins of the NF-κB signal transduction pathway, like Iκ-Bα.

**Fig. 6:**
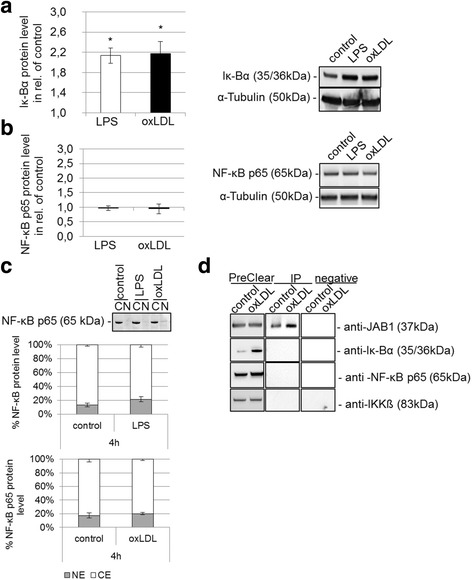
OxLDL-induced JAB1 and NF-κB signaling in human MФ. PMA-differentiated human U937 MΦ were treated (4 h) with or without 50 μg/ml oxLDL or 0.1 μg/ml LPS. The protein expression level of (**a**) Iκ-Bα and (**b**) NF-κB p65 were determined using western blot analysis and quantified by ImageJ. Expression was normalized against α-tubulin. Bars represent mean ± SEM of 6 (**a**) or 3 (**b**) independent experiments. *p < 0.005 significance vs. control. **c** Cytosolic protein [C] and nuclear protein [N] were extracted and western blot analysis was performed for the NF-κB p65 subunit and quantified by ImageJ (Bars represent mean + SEM of 5 independent experiments). **d** Immunoprecipitation of JAB1 was carried out with anti-JAB1 [2A10.8] (GenTex) antibodies followed by immunoblot detection for JAB1/COPS5, IKKß, Iκ-Bα and NF-κB in PreClear cell lysates and immunoprecipitates (IP). The eluate from Control Agarose Resin served as negative control. The depicted blot is representative for 3 independent experiments

We found that Iκ-Bα was significantly (*p* < 0.005) 2.17 ± 0.24-fold increased on the protein level after oxLDL treatment (4 h) of differentiated human U937 MΦ (Fig. 6a). Also, there were no statistically significant differences in NF-κB p65 protein quantity (Fig. 6b) or nuclear translocation, as shown by western blot (Fig. 6c). As positive control for inflammation, LPS treatment (4 h) significantly (*p* < 0.005) increased the Iκ-Bα protein expression (2.13 ± 0.15-fold), but no statistically significant translocation of NF-kB p65 into the nucleus could be substantiated after LPS treatment for 4 h (Fig. 6c).

In addition, we tested for potential the interaction of JAB1 with IKKß, Iκ-Bα and NF-κB in untreated or oxLDL-treated human MΦ by co-immunoprecipitation (Fig. 6d). We clarified the cell lysate by using the Control Agarose Resin in order to reduce to reduce non-specific protein binding (PreClear cell lysate) (Fig. 6d). In the resulting eluate we detected neither JAB1 nor IKKß, Iκ-Bα and NF-κB p65 protein, implicating that the performed immunoprecipitation was specific for the antibody against JAB1 (Fig. 6d). However, the co-immunoprecipitation showed no interaction of JAB1 with IKKß, Iκ-Bα and NF-κB p65 (Fig. 6d).

### OxLDL-induced phosphorylation of p38 MAPK and interaction with JAB1 *in vitro* in human MΦ and in arteriosclerotic plaques

**Fig. 7:**
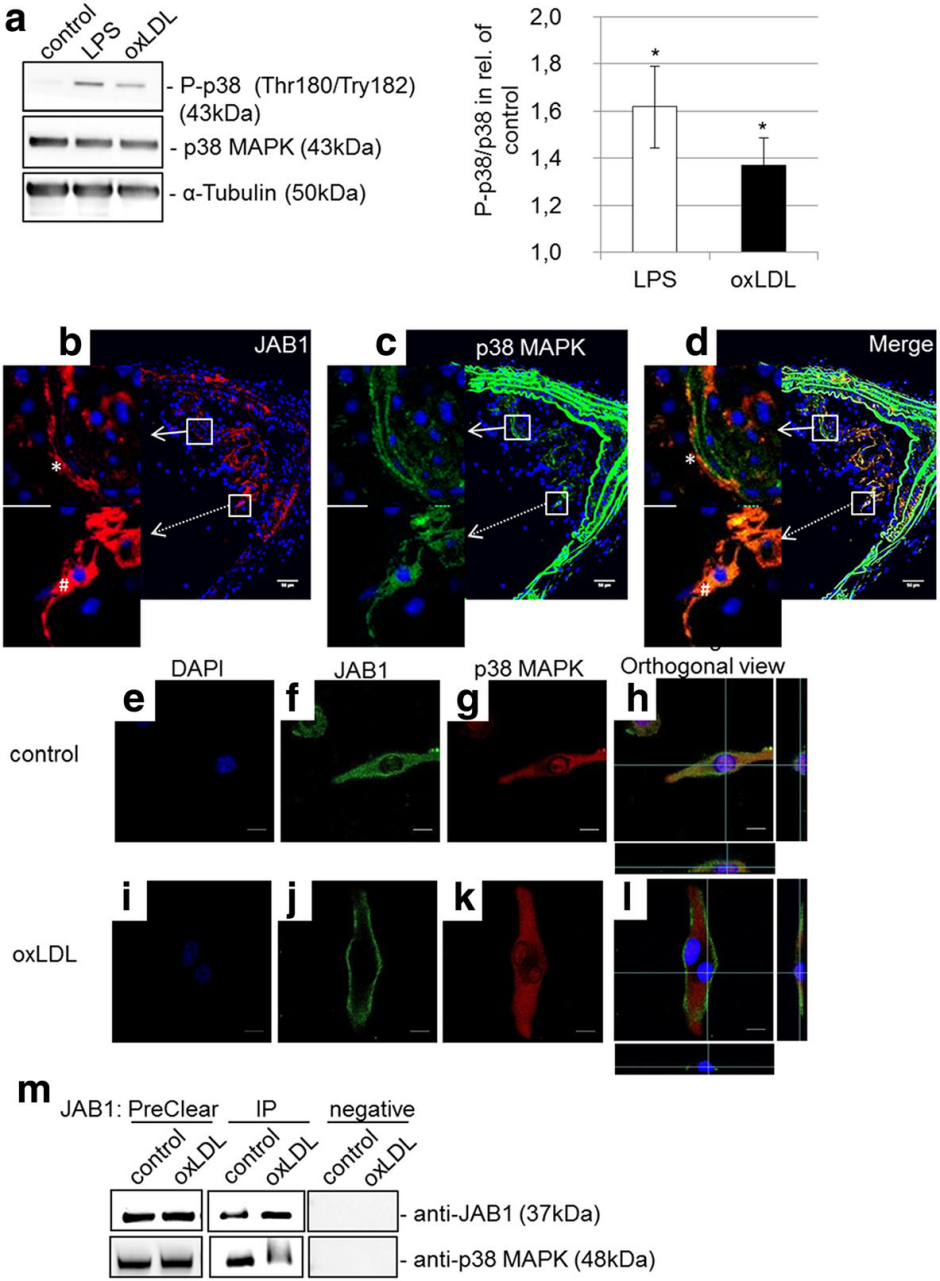
OxLDL-induced phosphorylation of p38 MAPK and interaction with JAB1 *in vitro* in human MΦ and in arteriosclerotic plaques. **a** The protein levels of p38 MAPK and P-p38 (Thr180, Try182) were determined by western blot analysis after treatment (4 h) of PMA-differentiated human U937 MΦ with or without 50 μg/ml oxLDL or 0.1 μg/ml LPS. Expression was normalized against α-tubulin. Bars represent mean ± SEM of 4 independent experiments (*p < 0.05). **b**–**﻿d** Representative cross-sections of the brachiocephalic trunk of ApoE^−/−^ mice at 30 weeks of age. JAB1 (**b**) is co-localized (broken arrow) and not co-localized (white arrow) with p38 MAPK (**c**) in atherosclerotic plaque, as seen in merge (**d**). JAB1 was detected on the cell surface/cell membrane (*) or in the cytosol (#) in atherosclerotic plaque (**b**, **d**). Immunofluorescent staining of JAB1 and p38 MAPK was observed by confocal microscopy (Nikon Eclipse) and analyzed with Fiji ImageJ. The individual channels are depicted in columns: blue, DNA; red, JAB1/COPS5; green, p38 MAPK. Representative photos from 4 independent experiments with similar results are shown. Bars: 50 μm (**b**, **c**, **d**). **e**–**l** Immunofluorescence of JAB1 (**f**, **j**) and p38 MAPK (**g**, **k**) in PMA-differentiated human U937 MΦ was observed by confocal laser scanning microscopy (Nikon Eclipse) and analyzed with Fiji ImageJ. The individual channels are depicted in columns: blue, DNA; green, JAB1/COPS5; red, p38 MAPK. (**h**, **l**) The white cross represents the intersection of x-, y- and z-axis in the orthogonal coordinate system. Representative immunofluorescence results from 3 independent experiments are shown. Scale bars: 10 μm. **m** Immunoprecipitation (IP) of JAB1 was carried out with anti-JAB1 [2A10.8] (GenTex) antibodies followed by immunoblot detection for JAB1 and p38 MAPK in PreClear cell lysates. The eluate from Control Agarose Resin served as negative control. The blot is representative for 4 independent experiments

The targets of the NF-kB and p38 MAPK signaling pathway are transcription factors that influence pro-inflammatory cytokines like IL-6 and TNF-α. We showed that independent from NF-kB signaling the TNF-α and IL-6 mRNA expression and protein secretion were significantly increased in oxLDL-treated MΦ in comparison to control MΦ. Therefore, we investigated the activation of p38 MAPK marked by the phosphorylation of Thr180 and Tyr182. LPS- or oxLDL-treatment (4 h) of differentiated human U937 MΦ induced the activation of p38 MAPK as indicated by a significantly increased protein expression (37% ± 11% or 62% ± 17%; *p* < 0.05) of phosphorylated p38 MAPK (Fig. 7a). In addition, we analyzed the co-localization and interaction of JAB1 with p38 MAPK *in vivo* and *in vitro*. Our immunohistochemical investigations showed that p38 MAPK-ir cells were predominantly localized in shoulder and middle regions of atherosclerotic plaques of ApoE^−/−^ mice (Fig. 7b-d). Additionally, using double immunofluorescence technique, p38 MAPK-ir was partly co-localized and regionally not co-localized with JAB1-ir in the shoulder region of the plaque (Fig. 7b-d). We found JAB1 co-localized with p38 MAPK in the cytosol or not co-localized with p38 MAPK on the cell surface/in the cell membrane in an atherosclerotic plaque of ApoE^−/−^ mice (Fig. 7b, d).

Furthermore, we analyzed the co-localization and interaction of JAB1 with p38 MAPK without oxLDL and after oxLDL treatment using immunofluorescence confocal laser scanning microscopy (Fig. 7e-l) or co-immunoprecipitation (Fig. 7m). We found an interaction of JAB1 with p38 MAPK in untreated (= negative control) human MΦ, but fewer after 4 h oxLDL exposition (Fig. 7i-m). In the eluate of the Control Agarose Resin (negative control), we detected neither JAB1 nor p38 MAPK protein, indicating successful immunoprecipitation specific for the antibody against JAB1 (Fig. 7m). Quantification of co-immunoprecipitated (CO-IP) JAB1 by Western Blot analysis in the eluate revealed a significant increase (20%; *p* = 0.02 vs. control) of JAB1-protein level in comparison with the control (data not shown). Using confocal laser scanning microscopy, we found that in untreated (=negative control) MΦ JAB1 immunofluorescence was detected mainly in the cytosol (Fig. 7f). After oxLDL treatment, however, JAB1 immunofluorescence was located on the cell surface/in the cell membrane (Fig. 7j). Moreover, p38 MAPK was found in the cytosol and in the nucleus of untreated and oxLDL-treated human MΦ (Fig. 7g, k). Furthermore, JAB1 and p38 MAPK immunofluorescence signals were co-localized in the cytosol in control, but not in oxLDL-treated human MΦ (Fig. 7h, l).

## Discussion

Blood cholesterol and particularly LDL cholesterol, especially oxLDL, plays a key role in all stages of atherosclerosis by regulating the expression of adhesion molecules, chemokines and pro-inflammatory cytokines. Moreover, oxLDL triggers processes mediated by various signaling pathways in different cell types, such as recruitment of mononuclear cells, lipid accumulation in MΦ (foam cell formation) as well as inflammatory and cytotoxic responses [28, 29]. Additionally, oxLDL plays a central role in the genesis of vulnerable plaques and plaque instability in human coronary arteries with lesions due to abundant foam cell formation [30].

Here we showed a localization of JAB1 in CD68+ MΦ in atherosclerotic lesions of the brachiocephalic trunk of hypercholesterolemic ApoE^−/−^ mice (after 20 weeks CED). In non-atherosclerotic/plaque-free arteries JAB1-ir was not observed. These data indicate an important role of JAB1 during foam cell formation and consecutive atherosclerotic plaque development and progression. Our data of CD68-ir MΦ in atherosclerotic lesions are in accordance with other reports describing JAB1 positive CD68-ir MΦ in human aortic fatty streak lesions [11]. Moreover, it has been described that different stages of human atherosclerosis contain different amounts of foam cells, associated with JAB1 expression [11] and athero-progression [3]. For this reason, our data and those of others suggest that JAB1 is involved in the ubiquitin-proteasome degradation machinery [16] and participates in the regulation of signal transduction pathways [10, 20] concerning oxLDL internalization/metabolization.

Based on the co-localization of JAB1- and CD68^+^-ir in MΦ of atherosclerotic lesions, e.g. of the brachiocephalic trunk of ApoE^−/−^ mice, we examined the effect of oxLDL on foam cell formation *in vitro* and the role of JAB1 in this process, which is still unclear. Here we showed that treatment of differentiated human MΦ with oxLDL resulted in foam cell formation *in vitro* with a significant and characteristic intracellular storage of lipid-droplets, as well as a significant increased content of TC, FC and EC. In contrast, treatment with LPS, as a positive control for inflammation, did not lead to foam cell formation. These data are in accordance with others using human U937 MΦ [31, 32] or murine RAW 264.7 [7]. Moreover, we found a significant increase of JAB1 protein expression of more than 50% in human foam cells after oxLDL incubation - and to a lower percentage (~30%; not significant) also after LPS treatment. These data indicate that oxLDL induces JAB1 expression *in vivo* and *in vitro*. However, JAB1 does not directly regulate the cellular cholesterol content in human MФ, as demonstrated e. g. by our JAB1-siRNA experiments. Therefore, this regulation may be due to COP9, containing JAB1 as a subunit, that controls the ubiquitinylation of ATP-binding cassette protein A1 (ABCA1), which mediates the transfer of cellular free cholesterol and phospholipids to apolipoprotein A-1 (apoA-1) [33].

Given that oxLDL plays a key role in all stages of atherosclerosis by regulating the expression of JAB1 and pro-inflammatory cytokines, we showed an oxLDL-induced upregulation of TNF-α and IL-6 mRNA expression and protein secretion in differentiated human MФ. Vice versa, JAB1 silencing (up to 60% on mRNA expression and protein level in oxLDL stimulated human MФ) resulted in a significant decrease of TNF-α and IL-6 mRNA expression and protein secretion after oxLDL treatment in comparison to nsiJAB1-MФ. These data indicate that the oxLDL-induced expression and secretion of these two pro-inflammatory cytokines depend on JAB1 expression in human MФ. Furthermore, it has been reported most recently that the genetic inhibition of JAB1 using knock-down gene expression abrogated the binding of the inhibitory Iκ-Bα subunit to NF-κB and enhanced TNF-α production in EC, while JAB1 overexpression had the opposite effect, suggesting a crucial role of JAB1 after TNF-α stimulation [10]. However, our study cannot confirm these findings in oxLDL treated human MФ, possibly due to different cell types and/or different treatment.

Moreover, we tested the effects of oxLDL on Ik-Bα and NF-κB p65 expression in differentiated human MΦ, since the ubiquitination machinery necessary to activate the NF-kB p65 might be regulated by the COP9 signalosome [10, 18]. Our present data demonstrate that oxLDL treatment of differentiated human MΦ resulted in an increased Iκ-Bα protein level. Additionally, we observed no significant nuclear translocation of NF-κB p65 after 4 h incubation of human MΦ with oxLDL. However, the NF-ĸB p65 nuclear translocation seems to be time-dependent after oxLDL or LPS stimulation of human MФ, because we and others found a NF-ĸB p65 nuclear translocation after 24 h oxLDL or LPS treatment (see Additional file 5) [34]. Therefore, it seems that JAB1 does not influence the NF-kB p65 signaling after 4 h oxLDL treatment in differentiated human MΦ.

Furthermore, the p38 pathway is implicated in cholesterol ester accumulation in MΦ and the development of atherosclerosis [35], since its activation plays an essential role in the production of inflammatory cytokines (TNF-α, IL-6) [36]. It has already been demonstrated that JAB1 is critically involved in TLR-mediated activation of p38 MAPK and ERK in mouse MФ [20]. We here show that JAB1 is an important factor for p38 MAPK mediated inflammation, however obviously independent from NF-kB in human MФ after oxLDL treatment. Therefore, future studies are necessary to prove the molecular mechanism of JAB1 and p38 MAPK in oxLDL treated MФ. In our experiments, stimulation of differentiated human MΦ with oxLDL led to significant increases of the phosphorylated p38 (P-p38) and mRNA expression of the pro-inflammatory cytokines TNF-α and IL-6, accompanied by an increase of the intracellular lipid accumulation. Previous studies with murine RAW264.7 MΦ showed that the inhibition of p38 MAPK with curcuma, a naturally occurring yellow pigment from the rhizomes of the plant *Curcuma longa* L., leads to the inhibition of oxLDL-induced foam cell formation and of CD36 expression [7]. Our results extend the importance of p38 MAPK activation during oxLDL-induced foam cell formation in human MФ and for the production of pro-inflammatory mediators such TNF-α and IL-6 [37, 38] implicating a major role during atherosclerotic processes.

Furthermore, we identified the relationship between the oxLDL-induced p38 activation, increase of JAB1 protein level and foam cell formation. In addition, we showed a loss of co-localization and interaction of JAB1 with p38 MAPK by confocal laser scanning immunofluorescence technique and co-immunoprecipitation after 4 h oxLDL treatment. We showed *in vivo* that JAB1 was not co-localized with p38 MAPK and localized on the cell surface/in the cell membrane in some parts within the shoulder regions of atherosclerotic lesions of ApoE^−/−^ mice, indicating possible inflammatory processes in areas prone to rupture. In addition, we observed a translocation of JAB1 from the cytosol to the cell membrane in U937 MФ after 4 h oxLDL treatment. Particularly in this context, we identified that oxLDL treatment leads to p38 MAPK activation through phosphorylation at the sites Thr180 and Try182 by the elimination of JAB1 from p38 MAPK. The activation of p38 MAPK signaling pathway leads to increase expression of cytokines (TNF-a, IL-6) and JAB1 protein. The JAB1 silencing resulted in decrease of cytokine expression and secretion. JAB1 up-regulates the innate immune response by activation of MAPK-mediates pathway. On the other side, our data corroborate the hypothesis that JAB1 is regulated by MAPK-mediates pathway. However, another study showed that JAB1 deletion significantly attenuates the induction of phosphorylated p38 and ERK upon TLR ligand stimulation in bone marrow-derived mouse MΦ [20]. This attenuation is specific for p38 MAPK and ERK without affecting activation of NF-ĸB, and related to a possible inflammatory role of JAB1 [20]. Further research is needed to understand why JAB1 silencing leads to prevention of inflammation and also what are the functional background of the interaction of JAB1 and p38 MAPK in human MФ.

The recent data indicate that JAB1 is critically involved in the oxLDL-induced inflammatory signaling pathway and cytokine secretion (TNF-α, IL-6), with potential consequences for atherosclerotic plaque progression and plaque rupture.

## Conclusion

In summary, we showed that JAB1, which can be stimulated by LPS or oxLDL, acts as a mediator between different inflammatory signaling pathways in MΦ during oxLDL exposition, without influencing the foam cell formation under our experimental conditions. Further investigations are required to understand the impact of oxLDL and p38 MAPK on JAB1 regulation, cellular localization and the pivotal participation in plaque progression. In this context, our data provide plausible evidence to consider the JAB1 subunit of COP9 as a possible target for the treatment of diseases associated with chronic inflammation and particularly with atherosclerosis.

## Additional files

Additional file 1: Antibodies used in this study. (PDF 146 kb)
Additional file 2: Primers used in this study. (PDF 134 kb)
Additional file 3: Figure S1. OxLDL influence JAB1 protein expression and foam cell formation in differentiated human MΦ. (PDF 258 kb)
Additional file 4: Knock-down of JAB1 in human MФ. (PDF 264 kb)
Additional file 5: OxLDL-induced NF-κB signaling in human MФ after 24 h. (PDF 340 kb)

## Abbreviations

ABCA1: ATP-binding cassette protein A1
ANOVA: Analysis of variance
apoA-1: Apolipoprotein A-1
apoE: Apolipoprotein E
BCA: Bicinchoninic acid
CCL2: C-C motif chemokine ligand 2
CE: Cholesteryl ester
CED: Cholesterol-enriched diet
COP9: Constitutive photomorphogenesis 9
CRL: Cullin-RING ubiquitin ligase
CSN5: COP9 signalosome complex unit 5
FC: Free cholesterol
HUVEC: Human umbilical vein endothelial cells
ICAM-1: Intercellular adhesion molecule 1
IKKß: IkB kinase ß
IL-6: Interleukin 6
ir: Immunoreactive
Iκ-Bα: Nuclear factor of kappa light polypeptide gene enhancer in B-cells inhibitor, alpha
JAB1: Jun activation domain binding protein-1
JAMM: JAB1/MPN domain metalloenzyme
LPS: Lipopolysaccharide
MAPK: Mitogen-activated protein kinase
MK2: MAPK-activated protein kinase-2
MPN: Mpr/Pad1/N-terminal
MΦ: Macrophages
NF-κB: Nuclear factor-kappa B
nsiJAB1: Negative control siJAB1
OD: Optical density
ORO: Oil red O
oxLDL: Oxidized low-density lipoprotein
PBS: Phosphate buffered saline
PMA: Phorbol 12-mystriate 13-acetate
RIPA: Radio-immunoprecipitation assay
SCF: Skp1, Cullins, F-box proteins
SEM: Standard error of the mean
siJAB1: siRNA JAB1
TBS: Tris buffered saline
TC: Total cholesterol
TNF-α: Tumor necrosis factor-alpha
UPS: Ubiquitin proteasome system
VCAM-1: Vascular cell adhesion molecule 1
