# Supplementary material for: Oxidized LDL-induced JAB1 influences NF-κB independent inflammatory signaling in human macrophages during foam cell formation
Source: J Biomed Sci. 2017 Feb 7;24:12. doi: 10.1186/s12929-017-0320-5 (PMC5297127; doi:10.1186/s12929-017-0320-5)
Supplement: Additional file 1: — Antibodies used in this study. (PDF 146 kb) [file 12929_2017_320_MOESM1_ESM.pdf]

Additional file 1

Antibodies used in this study.

Antigen	Antibody name	Cat. no.	Company
Alpha Tubulin	Polyclonal rabbit anti- α-Tubulin	ab4074	Abcam plc., Cambridge, UK
CD68	monoclonal rat anti-CD68	MCA1957	AbD Serotec, Kidlington, UK
Iκ-Bα [E130]	monoclonal rabbit anti-Ik-Bα [E130]	ab32518	Abcam plc., Cambridge, UK
JAB1[2A10.8]	Monoclonal mouse anti-JAB1[2A10.8]	GTX70203	GeneTex, Irvine, CA, USA
NF-κB p65 (C-20)	polyclonal goat anti- NF-kB p65 (C-20)	sc-372-G	Santa Cruz Biotechnology Inc.; Dallas, USA
p38 MAPK	Polyclonal rabbit anti-p38 MAPK	9212	
Phospho-p38 MAPK (Thr180/ Try182) (28B10)	Monoclonal mouse anti-Phospho-p38 MAPK (Thr180/ Try182) (28B10)	9216	Cell signaling, Cambridge, UK
IKKß (D30C6)	Monoclonal rabbit anti-IKKß (D30C6)	8943	
**Secondary Antibody**			
Goat-anti-mouse IgG (H+L)-Cy2		115-225-003	
Goat-anti-mouse IgG (H+L)-Cy3		115-165-146	Dianova GmbH, Hamburg, Germany
Goat-anti-rabbit IgG (H+L)-Cy3		111-165-003	
Goat-anti-rat IgG HRP-Cy3		STAR72	AbD Serotec, Kidlington, UK
