# Supplementary material for: Oxidized LDL-induced JAB1 influences NF-κB independent inflammatory signaling in human macrophages during foam cell formation
Source: J Biomed Sci. 2017 Feb 7;24:12. doi: 10.1186/s12929-017-0320-5 (PMC5297127; doi:10.1186/s12929-017-0320-5)
Supplement: Additional file 2: — Primers used in this study. (PDF 134 kb) [file 12929_2017_320_MOESM2_ESM.pdf]

Additional file 2

Primers used in this study.

Gene symbol	Detected transcript	amplicon length	Cat. no.:
**Reference Genes**			
RPLP0	NM_001002, NM_053275	170bp	QT01839887
TBP	NM_001172085, NM_003194	132bp	QT00000721
B2M	NM_004048	98bp	QT00088935
**Genes of Interest**			
TNF-α	NM_000594	98bp	QT00029162
IL6	NM_000600	107bp	QT00083720
