# Supplementary material for: Oxidized LDL-induced JAB1 influences NF-κB independent inflammatory signaling in human macrophages during foam cell formation
Source: J Biomed Sci. 2017 Feb 7;24:12. doi: 10.1186/s12929-017-0320-5 (PMC5297127; doi:10.1186/s12929-017-0320-5)
Supplement: Additional file 3: Figure S1. — OxLDL influence JAB1 protein expression and foam cell formation in differentiated human MΦ. (PDF 258 kb) [file 12929_2017_320_MOESM3_ESM.pdf]

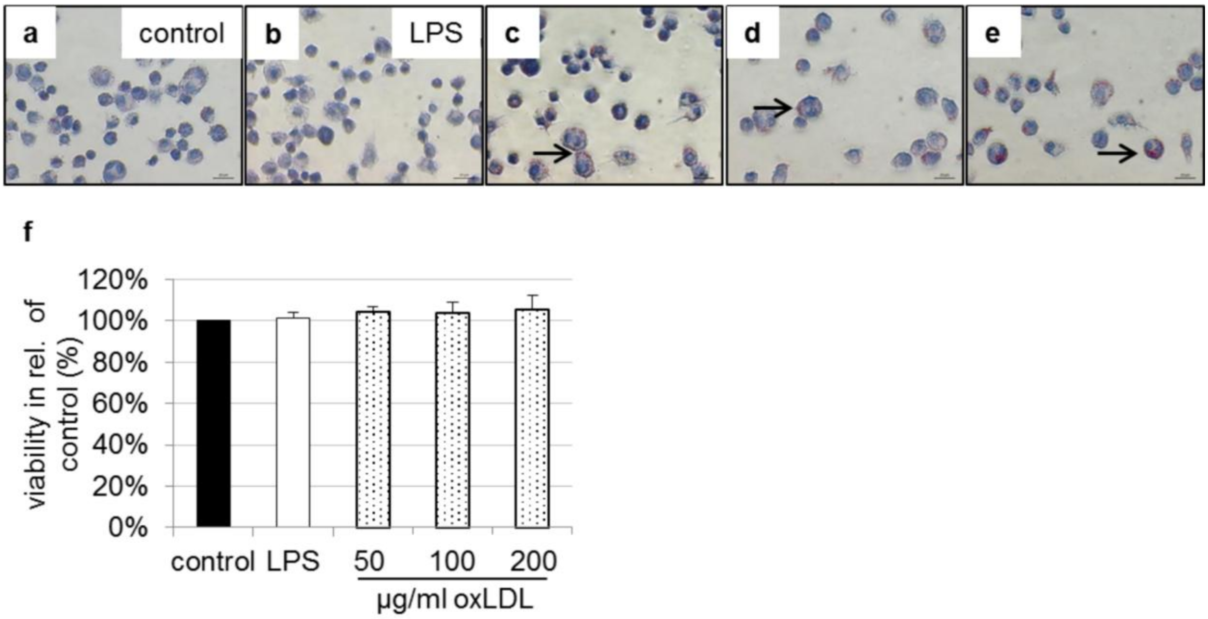

Figure S1: OxLDL influence JAB1 protein expression and foam cell formation in differentiated human MΦ. (a – e) PMA-differentiated human U937 MΦ were incubated (4h) with (a) medium alone (= control), (b) 0.1µg/ml LPS, (c) 50 µg/ml, (d) 100 µg/ml or (e) 200 µg/ml oxLDL. Cells were stained with OilRedO (ORO) and evaluated by interference light microscopy. Black arrows indicate cells containing lipid droplets identified by ORO. Bars: 20µm. (f) Viability of MΦ after incubation (4h) with oxLDL (50 µg/ml, 100 µg/ml, 200 µg/ml) or LPS. Values (in % viability of cells without treatment [=control; =100% viability]) are given as mean + SEM; n=3 independent experiments.
