# Supplementary material for: Oxidized LDL-induced JAB1 influences NF-κB independent inflammatory signaling in human macrophages during foam cell formation
Source: J Biomed Sci. 2017 Feb 7;24:12. doi: 10.1186/s12929-017-0320-5 (PMC5297127; doi:10.1186/s12929-017-0320-5)
Supplement: Additional file 4: — Knock-down of JAB1 in human MФ. (PDF 264 kb) [file 12929_2017_320_MOESM4_ESM.pdf]

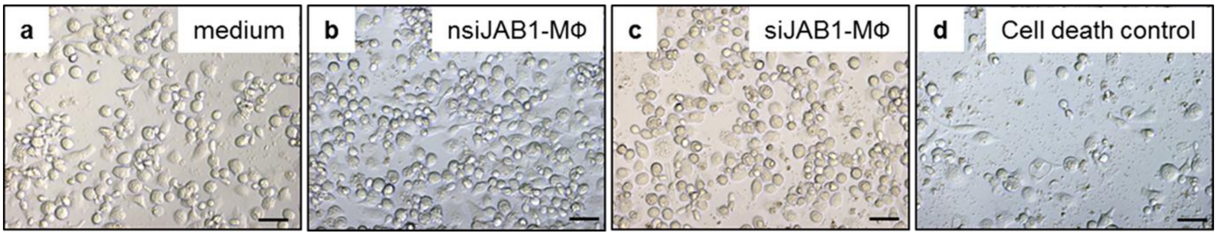

Additional file 4: Knock-down of JAB1 in human MΦ. PMA-differentiated human U937 MΦ were treated with HiPerfect Transfection Reagent (medium) (a), transfected with negative siRNA (nsiJAB1-MΦ) (b), siRNA JAB1 (siJAB1-MΦ) (c), or positive siRNA (cell death control) (d) for 48h. Representative images results from 5 independent experiments are shown. Bars: 20µm
