# Supplementary material for: Oxidized LDL-induced JAB1 influences NF-κB independent inflammatory signaling in human macrophages during foam cell formation
Source: J Biomed Sci. 2017 Feb 7;24:12. doi: 10.1186/s12929-017-0320-5 (PMC5297127; doi:10.1186/s12929-017-0320-5)
Supplement: Additional file 5: — OxLDL-induced NF-κB signaling in human MФ after 24 h. (PDF 340 kb) [file 12929_2017_320_MOESM5_ESM.pdf]

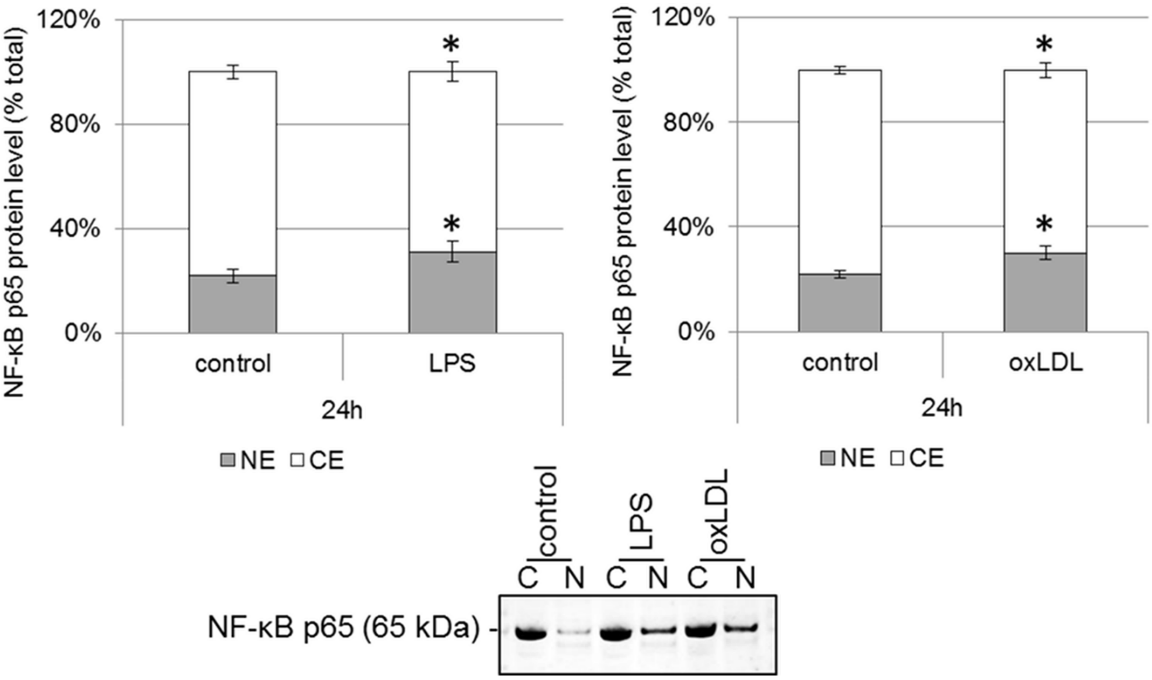

Additional file 5: OxLDL-induced NF-κB signaling in human MΦ after 24h. PMA-differentiated human U937 MΦ were treated (4h) with or without 50μg/ml oxLDL or 0.1μg/ml LPS. Cytosolic protein [CE, C] and nuclear protein [NE, N] were extracted and western blot analyses were performed for the NF-κB p65 subunit and quantified by ImageJ (Bars represent mean + SEM of 5 independent experiments). *P<0.05 vs control.
